# MinIONQC: fast and simple quality control for MinION sequencing data

**DOI:** 10.1093/bioinformatics/bty654

**Published:** 2018-07-23

**Authors:** R Lanfear, M Schalamun, D Kainer, W Wang, B Schwessinger

**Affiliations:** The Research School of Biology, Australian National University, Canberra ACT 2601, Australia

## Abstract

**Summary:**

MinIONQC provides rapid diagnostic plots and quality control data from one or more flowcells of sequencing data from Oxford Nanopore Technologies’ MinION instrument. It can be used to assist with the optimisation of extraction, library preparation, and sequencing protocols, to quickly and directly compare the data from many flowcells, and to provide publication-ready figures summarising sequencing data.

**Availability and implementation:**

MinIONQC is implemented in R and released under an MIT license. It is available for all platforms from https://github.com/roblanf/minion_qc.

## 1 Introduction

Oxford Nanopore Technologies’ (ONT) small and portable MinION instrument is revolutionising DNA sequencing. It allows users to go from sample to sequence in hours, it can sequence extremely long DNA molecules, and it provides many gigabases of data from each flowcell. Because of this, many research groups and companies are adopting the instrument for in-house and in-field sequencing.

Here we present MinIONQC: a fast, lightweight, and non-interactive script to provide quality control and diagnostic analyses of sequencing data from the MinION. MinIONQC differs from related tools (De Coster *et al.*, 2018; Loman and Quinlan, 2014; Stewart and Watson, 2017; Watson *et al.*, 2015) in that it is focussed primarily on the rapid and replicable comparison of large volumes of sequencing data from multiple flowcells. MinIONQC will assist with cases where the rapid and repeated comparison of data from multiple flowcells is required, including the application of MinION sequencing in new use cases (e.g. with new tissues or in new settings), and in completing large genome projects which require the aggregation of data from many flowcells (Austin *et al.*, 2017; Jain *et al.*, 2017; Jansen *et al.*, 2017; Schmidt *et al.*, 2017; Tan *et al.*, 2018).

## 2 Software description

MinIONQC is written in R and designed to be run non-interactively from the command line. This facilitates automation of the script on all platforms, including in bioinformatics pipelines run on remote servers. MinIONQC is packaged as a single lightweight script that will work on all platforms that run R. It requires minimal installation and has just a small number of dependencies that can be installed in under a minute (Davis, 2018; Dowle and Srinivasan, 2018; Garnier, 2018; Lee and Rowe, 2016; Stephens *et al.*, 2018; Wickham, 2007, 2009, 2011, 2017; Wickham *et al.*, 2017). It has extensive documentation, a full test suite, and example input and output files available at https://github.com/roblanf/minion_qc. On a standard desktop computer with four processors, it is capable of analysing output from 24 flowcells, which produced a combined 107GB of sequencing data, in 25 min.

## 3 Quality control of a single flowcell

For each flowcell, MinIONQC outputs a human- and machine-readable summary file in YAML format. This file contains information on the total number of sequenced bases and reads, as well as a number of widely-used statistics of read lengths and quality scores, including the number of reads and bases from ‘ultra-long’ reads, defined as the largest set of reads with an N50 greater than 100 KB (Jain *et al.*, 2017). All statistics are calculated for the complete dataset, as well as for the subset of reads that pass a user-defined quality score cutoff.

**Fig. 1.**
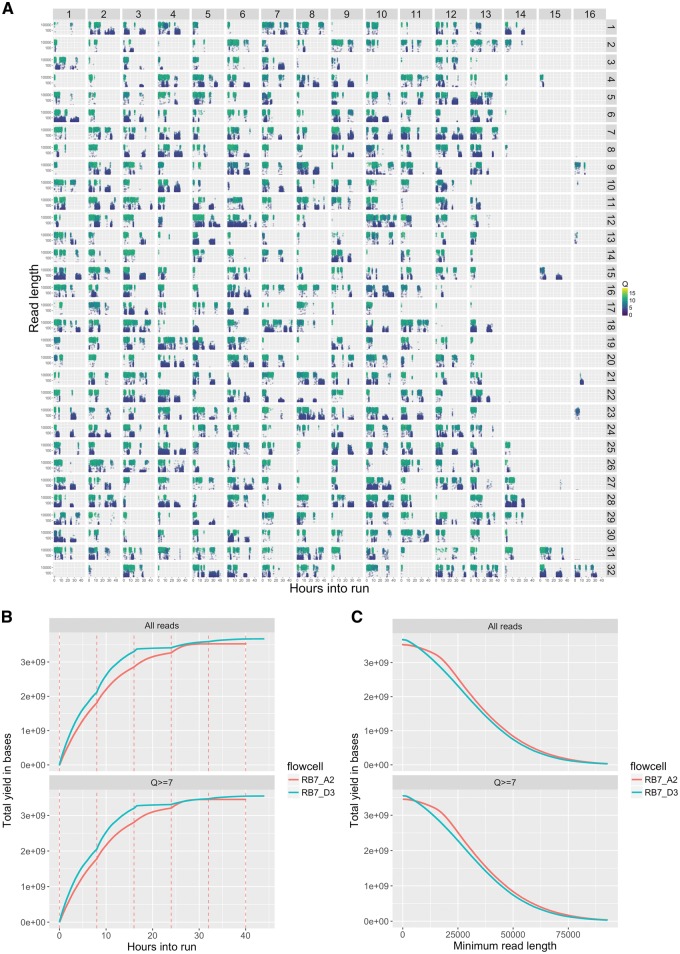
Three example plots from MinionQC. (**A**) A physical map of the flowcell with each of the 512 pores shown in their physical location. The sub-plot for each pore shows a single point for each read, with the length on the *y* axis (log scale), the number of hours into the run on the *x*-axis, and the quality score of the read as the colour. This plot clearly shows the presence of a bubble causing many of the pores on the right-hand-side of the plot to produce little or no data, as well as the presence of contaminants blocking the pores, leading to the production of a large number of small, low-quality signals as the run progresses; (**B**) Yield in bases (*y*-axis) against run time (*x*-axis) for two flowcells (each in a different colour), with the yield of all reads shown in the upper panel, and the yield of reads with a mean *Q* score above the user-specified threshold of 7 in the lower panel, vertical red dashed lines indicate the timing of group changes (also known as muxes); (**C**) Yield in bases (*y*-axis) for a given minimum read length (*x*-axis), for two flowcells (each in a different colour), panels are as in B (Color version of this figure is available at *Bioinformatics* online.)

MinIONQC produces ten plots for each flowcell. These include standard plots such as the distributions of read lengths and quality scores, the number of reads generated per hour, and the total yield of bases over time. MinIONQC also produces plots designed to assist with optimisation of laboratory procedures for subsequent sequencing runs such as a physical map of the flowcell including every sequenced read, which facilitates rapid diagnosis of common issues such as bubbles introduced during library loading, and the presence of contaminants which block pores on the flowcell during sequencing (Fig. 1A).

## 4 Comparing and combining data from multiple flowcells

Many projects, such as those that seek to assemble large or repeat-rich genomes, require the aggregation of data from many flowcells. MinIONQC simplifies the assessment of such data by allowing users to run the script on a single parent directory that contains multiple ‘sequencing_summary.txt’ files (produced by ONT’s Albacore and Guppy basecallers) in sub-directories. The resulting diagnostics simplify the management of larger projects by making it easy to assess the point at which sufficient data have been generated to move from sequencing to downstream analyses such as genome assembly.

MinIONQC produces two kinds of plots when given multiple flowcells as input: plots of the combined data that are directly comparable to those produced for a single flowcell (see above); and plots designed to compare the flowcells to each other. The six comparison plots include distributions of read lengths and quality scores, the changes in both quantities over the course of each sequencing run, the total yield of bases over time (Fig. 1B), and the total yield of bases by minimum read length (Fig. 1C). The latter plot is particularly useful in comparing the effects of different DNA extraction, cleanup, and library-preparation methods on the final distribution of read lengths. For example, Figure 1C shows data from one flowcell (RB7_A2, in red) in which DNA was size-selected using a Blue Pippin instrument, and another (RB7_D3, in blue) in which DNA was size selected using a bead-based protocol (Schalamun and Schwessinger, 2017). Both approaches produced similar total yields of high-quality reads (roughly 3.5 gigabases, as shown by the point at which each line in Fig. 1C crosses the *y*-axis) but the yield of reads greater than 20KB was clearly higher when using the Blue Pippin, as shown by the red line in Figure 1C being higher than the blue line at a value of 20KB on the *x* axis.

## 5 Conclusion

MinIONQC is a fast and efficient script to analyse the output from ONT’s MinION instrument. We hope that it will be useful to the community, and will facilitate further improvements and developments in the ways that the MinION is used.

## Funding

This work was supported by Australian Research Council grants to R.M.L and B.S.

*Conflict of Interest*: none declared.

## References

[bty654-B1] Austin C.M. et al (2017) De novo genome assembly and annotation of Australia's largest freshwater fish, the Murray cod (Maccullochella peelii), from Illumina and Nanopore sequencing read. Gigascience, 6, 1. 6. 10.1093/gigascience/gix063 PMC5597895 28873963

[bty654-B2] Davis T.L. (2018) Optparse: Command Line Option Parser. R Package Version 1.6.0. CRAN: the Comprehensive R Archive Network.

[bty654-B3] De Coster W. et al (2018) NanoPack: visualizing and processing long read sequencing data. Bioinformatics, 25, 1422. 10.1093/bioinformatics/bty149 PMC6061794 29547981

[bty654-B4] Dowle M., Srinivasan A. (2018) *Data.table: extension of ‘Data.frame’ R Package Data.table Version 1.11.4 CRAN: The Comprehensive R Archive Network.*

[bty654-B5] Garnier S. (2018) Viridis: default Color Maps from ‘matplotlib’ R Package Version 0.5.1. CRAN: the Comprehensive R Archive Network.

[bty654-B6] Jain M. et al (2017) Nanopore sequencing and assembly of a human genome with ultra-long reads. Nat. Biotechnol., 1, 74. 10.1038/nbt.4060 PMC5889714 29431738

[bty654-B7] Jansen H.J. et al (2017) Rapid de novo assembly of the European eel genome from nanopore sequencing reads. Sci. Rep., 7, 7213. 28775309 10.1038/s41598-017-07650-6 PMC5543108

[bty654-B8] Lee B., Rowe Y. (2016) futile.logger: A Logging Utility for R. R package version 1 the Comprehensive R Archive Network. **4**, 3. CRAN.

[bty654-B9] Loman N.J., Quinlan A.R. (2014) Poretools: a toolkit for analyzing nanopore sequence data. Bioinformatics, 30, 3399–3401. 25143291 10.1093/bioinformatics/btu555 PMC4296151

[bty654-B10] Schalamun M., Schwessinger B. (2017) *DNA Size Selection (>1kb) and Clean up Using an Optimized SPRI Beads Mixture. protocols.io*.

[bty654-B11] Schmidt M.H.W. et al (2017) De novo assembly of a new Solanum pennellii accession using nanopore sequencing. Plant Cell, 29, 2336–2348. 29025960 10.1105/tpc.17.00521 PMC5774570

[bty654-B12] Stephens J. et al (2018) Yaml: Methods to Convert R Data to YAML and Back. R Package Version 2.1.19. CRAN: the Comprehensive R Archive Network.

[bty654-B13] Stewart R.D., Watson M. (2017) poRe GUIs for parallel and real-time processing of MinION sequence data. Bioinformatics, 33, 2207–2208. 28334395 10.1093/bioinformatics/btx136 PMC5870607

[bty654-B14] Tan M.H. et al (2018) Finding Nemo: hybrid assembly with Oxford Nanopore and Illumina reads greatly improves the clownfish (Amphiprion ocellaris) genome assembly. Gigascience, 7, 1–6. 10.1093/gigascience/gix137 PMC5848817 29342277

[bty654-B15] Watson M. et al (2015) poRe: an R package for the visualization and analysis of nanopore sequencing data. Bioinformatics, 31, 114–115. 25173419 10.1093/bioinformatics/btu590 PMC4271141

[bty654-B16] Wickham H. (2009) ggplot2: Elegant graphics for data analysis Springer, New York.

[bty654-B17] Wickham H. (2007) Reshaping Data with the reshapePackage. J. Stat. Software, 21, 1–20.

[bty654-B18] Wickham H. (2017) Scales: Scale Functions for Visualization. R Package Version 0.5.0. CRAN: the Comprehensive R Archive Network.

[bty654-B19] Wickham H. (2011) The split-apply-combine strategy for data analysis. J. Stat. Software, 40, 29.

[bty654-B20] Wickham H. et al (2017) Readr: Read Rectangular Text Data. R Package Version 1.1.1. CRAN: the Comprehensive R Archive Network.

